# Safety Evaluation and Experimental Study of a New Bionic Muscle Cable-Driven Lower Limb Rehabilitation Robot

**DOI:** 10.3390/s20247020

**Published:** 2020-12-08

**Authors:** Yan Lin Wang, Ke Yi Wang, Kui Cheng Wang, Zong Jun Mo

**Affiliations:** College of Mechanical and Electrical Engineering, Harbin Engineering University, Harbin 150001, China; wangyanlin0513@21cn.com (Y.L.W.); wangkuicheng@hrbeu.edu.cn (K.C.W.); 2015071711@hrbeu.edu.cn (Z.J.M.)

**Keywords:** bionic muscle cable, lower limb rehabilitation robot, safety performance factor, safety, evaluation index

## Abstract

Safety is a significant evaluation index of rehabilitation medical devices and a significant precondition for practical application. However, the safety evaluation of cable-driven rehabilitation robots has not been reported, so this work aims to study the safety evaluation methods and evaluation index of cable-driven rehabilitation robots. A bionic muscle cable (BM cable) is proposed to construct a bionic muscle cable-driven lower limb rehabilitation robot (BM-CDLR). The working principle of the BM-CDLR is introduced. The safety performance factors are defined based on the mechanical analysis of the BM-CDLR. The structural safety evaluation index and the use safety evaluation index of the BM-CDLR are given by comprehensively considering the safety performance factors and a proposed speed influence function. The effect of the structural parameters of the elastic elements in the BM cable on the safety performance factors and safety of the BM-CDLR is analyzed and verified by numerical simulations and experimental studies. The results provide the basis for further study of the compliance control strategy and experiments of the human-machine interaction of the BM-CDLR.

## 1. Introduction

In recent decades, the aging speed of the global population has substantially increased [1,2]. The various natural and unnatural causes (for example: stroke, hemiplegia, nervous system diseases, car accidents, etc.) have led to a rapid increase in the number of patients with movement disorders of the limbs, and the natural degradation of the basic motion functions of the elderly and other reasons [3,4,5] have caused the quality of life of a large number of people to decline, which severely reduces the desire of patients with movement disorders to pursue a happy life. Moreover, there is a problem with the shortage of medical rehabilitation resources around the world. Therefore, the study of scientific and repeatable training rehabilitation equipment has important practical significance for the recovery of patients’ motion function [6].

The recovery of the movement functions of patients has become a research frontier in recent years. At present, rehabilitation robots for human limbs mainly include exoskeleton-type and cable-driven-type rehabilitation robots, and they have their performance advantages and characteristics [7,8,9]. However, compared with exoskeleton-type lower limb rehabilitation equipment, the cable-driven lower limb rehabilitation robot (CDLR) has the advantages of lighter mass, better human-machine compatibility, strong reconstruction ability, and so on [10,11,12]. These performance characteristics have attracted the interest of many rehabilitation engineering researchers, who have studied various CDLRs with different configurations. Zou YP et al. [13,14] designed a movable CDLR, in which two cables are attached to the ankle joint through two pulleys, and the other two cables are fixed at the knee joint through two pulleys. Wang Y.L. et al. [15,16] designed a lower limb gait training robot, with a rigid motion branch, driven by three cables. Barbosa, AM et al. [17] designed a CDLR for which the movable platform can be respectively driven by one to six cables, which can independently complete the training of the hip joint, knee joint, and ankle joint. Zhao T. and Zi B. et al. [18] designed a cable-driven parallel robot (CDPR) that can change the angle and height of the cable masts. Gallina, P. et al. [19,20] designed a variable radius drum mechanism for the cable robot.

Although the CDLR has the above advantages and performance characteristics, the cable can only provide positive tension force and have low stiffness characteristics, so that the CDLR may have a pseudo-drag phenomenon. The safety of patients and the control strategy have become the biggest challenges during the training process [19,21,22]. In order to ensure the safety of patients, the following problems of the CDLR have been studied. First of all, the designed mechatronic structures must safely support the patient [23]. The stability of the CDPR is an important performance metric to ensure the safety of patients. Stability evaluation methods have been proposed such as the Krasovsky method [24] and the force, position, and system stiffness properties of the CDPR [12,25], respectively. Secondly, motion trajectory planning is an important way to improve the motion performance of the CDLR. The dynamic motion trajectory planning of a CDPR using an improved quintic B-spline curve [26], the movement trajectory planning of the starting and braking process with a high-order polynomial interpolation method [27,28], and a method to avoid cable failure operation and any consequent motion of the end-effector [29] were proposed to obtain the safe position of the end-effector. In addition, in order to further improve the adaptability and safety of the CDLR, a pneumatic artificial muscle driving method has been introduced into the cable-driven rehabilitation devices [30,31,32]. Finally, the study of the control strategy is a direct way to achieve training tasks and ensure patient safety. Wang Y.L. et al. [33] studied a compound control strategy of the active and passive training mode for a CDLR to reduce the surplus force generated by the movement of the end-effector and interference. Zi B. [34] designed a two-level controller by combining the PID algorithm and fuzzy theory to improve the position accuracy of the end-effector and adjust the parameters of the PID controller in real-time. Plooij, M. et al. [35] defined the safety of an overground body weight support system, called the RYSEN, using difference between the power for horizontal and vertical movements and the system power to perform its working tasks.

The methods of ensuring and improving the safety of the CDLR system were studied using different ways and from different aspects in the above reports, but they did not give the safety evaluation methods for the CDLR from the natural characteristics. Therefore, a mechanical model of a bionic muscle cable is proposed based on the Hill muscle model. The bionic muscle cables (BM cables) are introduced into the CDLR configuration to construct a BM-CLDR. Based on the mechanical analysis of the BM-CLDR, the safety performance factors are defined. Considering the patient’s motion tolerance ability and training task requirements, a speed influence function is proposed. On this basis, the use safety evaluation index of the BM-CLDR is given. Finally, numerical simulations and test experiments are performed to verify the rationality of the evaluation method and to analyze the influence of the structural parameters of the elastic elements in the BM cable on the safety of the BM-CLDR. This lays the foundation for further research on the compliance control strategy and experiments of human-machine interaction. 

## 2. Methodology

### 2.1. BM-CDLR

**Figure 1:**
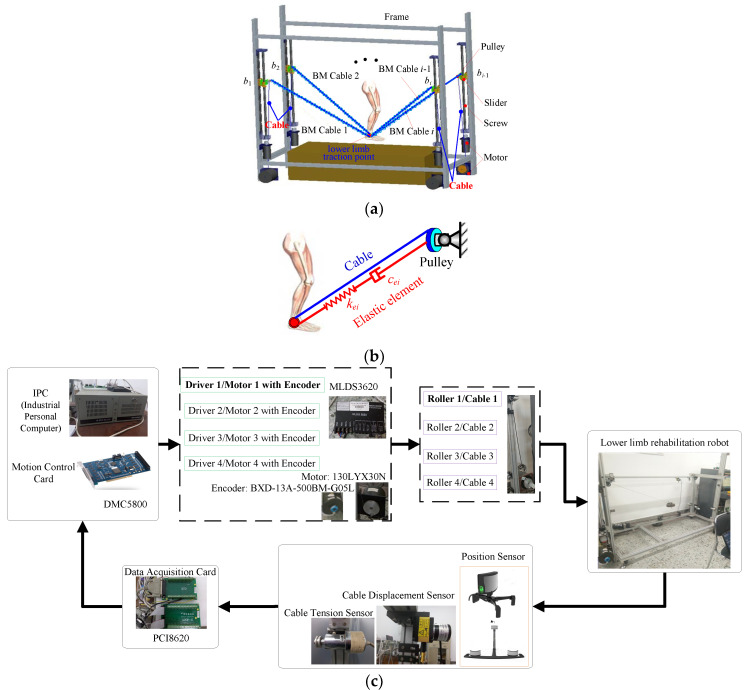
Bionic muscle (BM)-cable-driven lower limb rehabilitation robot (CDLR). (**a**) Structure of the BM-CDLR; (**b**) model of the BM cable; (**c**) block diagram of configuration for the BM-CDLR.

The CDPR has many applications in limb rehabilitation training. The performance requirements of the rehabilitation devices are relatively high in practical applications. However, the cable can only provide positive tension force, so that the pseudo-drag phenomenon may occur in the working process of the system, and this phenomenon will reduce the safety performance of the CDLRs. Therefore, a BM-CDLR is introduced in this paper, as shown in Figure 1. The BM-CDLR is 1.68 m long, 0.5 m wide, and 0.8 m high. 

The cable can only provide positive tension force for patients. When the external interference acts on the BM-CDLR system, the external interference may not be balanced in the direction of small restraint force to cause the motion of the lower limbs to deviate from the planned training task, which will reduce the training effect of the rehabilitation equipment, even causing the patient to be injured twice. In order to ensure the safety of patients and the training effect of the CDLR, based on the Hill muscle model, an elastic element is connected in parallel between the lower limb traction point *P* and the pulley *b_i_* to form a bionic muscle cable, as shown in Figure 1b. The existence of the elastic element will make the cable always in a tensional state, and the rehabilitation equipment will not have the pseudo-drag phenomenon. 

The configuration of the BM-CDLR is shown in Figure 1c. The BM-CDLR system is mainly composed of 4 groups of bionic muscle cable-driven units (BM-CDU), a treadmill, and a frame. The BM-CDU is composed of a BM cable and a pulley fixed on the slider, screw, tension sensors (Model: LDZL-08AF), and torque motor (Model: 130LYX30N) integrated photoelectric encoder (Model: BXD-13A-500BM-G05L) and the roller with a radius 40 mm. One end of the BM cable is attached to the ankle joint of the lower limb. The other end of the BM cable is connected to the roller fixed on the torque motor through the pulley. At the same time, one end of the elastic element in the BM cable is also connected to the ankle joint of the lower limb. The other end of the elastic element is connected to the pulley. 

In the actual training process, the height of the pulleys *b_i_* can first be set according to the height of the patients and the needs of the training task through the screw slider mechanism. Of course, the height of the pulleys *b_i_* can be adjusted in real-time during the training process according to the pre-planned training task. According to the planned training task, the IPC (Industrial Personal Computer) and the motion control card can generate control signals and input them into the drivers to obtain the desired motion of the BM-CDUs. The motion of the lower limb point can be realized by the coordinated motion control of the BM-CDUs. In addition, the data of the cable tension and the tracking position of the traction point, detected by the tension sensors and the position sensor, are fed back to the IPC to accurately or compliantly control the lower limb movement training, which can realize the closed-loop control of lower limb rehabilitation training.

The normal gait, running, and other training tasks can be achieved in active training mode, passive training mode, and auxiliary training mode by reasonably setting the running speed of the treadmill and the running mode of the BM-CDUs. In addition, the compound form rehabilitation training tasks of lower limbs in space can also be performed in the BM-CDLR system by the coordinating movement of the BM-CDUs, which includes the compound movement form trainings among the coronal plane, horizontal plane, and sagittal plane. 

### 2.2. Mechanical Analysis

As shown in Figure 1, in the global coordinate system *O*-*XYZ*, the position of the ankle joint of the lower limb traction point is ***P***(*x*, *y*, *z*). The position of the pulley ***b****_i_* is (*x_bi_*, *y_bi_*, *z_bi_*). The effective length of the BM cable is ***L****_i_*. Hence, the length vector of the BM cable can be expressed as:

(1) $$L_{i}=b_{i}-P\;(i=1,2,3,4)$$

The effective length of the BM cable can be expressed as:

(2) $$L_{i}=\sqrt{{\left(b_{i}-P\right)}^{T}\left(b_{i}-P\right)}$$

The lower limbs are driven by the cable tension force and the force generated by the elastic element to complete the desired movement. Therefore, the elastic element can be regarded as a “special cable” that can withstand the pressure force and the tension force. In addition, the elastic element can only provide the passive force, so the spinor corresponding to the force generated by the elastic element should be removed from the spinor matrix. The spinor corresponding to the force generated by the elastic element is regarded as the external force on the end-effector (i.e., traction point). Hence, the mechanical equilibrium equation of the BM-CDLR system can be written as:

(3) $$J^{T}T=J_{e}{}^{T}T_{e}+F$$

where $J^{T}$ denotes the structure matrix of the BM-CDLR. T $={\left[\begin{array}{cccc} t_{1} & t_{2} & t_{3} & t_{4} \end{array}\right]}^{T}$ denotes the cable tension vector and meets the condition $T_{\min}\leq T\leq T_{\max}$. $T_{\min}$ and $T_{\max}$ respectively indicate the minimum pre-tension force and the maximum allowable tension force of the cable. $J_{e}{}^{T}$ denotes the spinor matrix corresponding to the force generated by the elastic element in the BM cable. ***F*** is the resultant force of external force and gravity on the end-effector. $T_{e}$ $={\left[\begin{array}{cccc} t_{e1} & t_{e2} & t_{e3} & t_{e4} \end{array}\right]}^{T}$ is the force generated by the elastic element in the BM cable. It can be written as:

(4) $$t_{ei}=k_{ei}\left(L_{ei}-L_{e0}\right)-c_{ei}{\dot{L}}_{ei}$$

where $k_{ei}$, $L_{ei}$, $L_{e0}$, $c_{ei}$, and ${\dot{L}}_{ei}$ represent the stiffness coefficient, effective length, free length damping coefficient, and motion speed of the elastic element in the *i*th BM cable. Since the two ends of the elastic element are respectively connected to the traction point *P* and the pulley *b_i_*, the effective length and the motion speed of the elastic element in the *i*th BM cable meet the following relationship:

(5) $$\left\{ \begin{array}{l} L_{ei}=L_{i}\\ {\dot{L}}_{ei}={\dot{L}}_{i} \end{array}\right.$$

According to the mechanical balance Equation (3), the cable tension can be further expressed as:

(6) $$T=J^{+}\left(J_{e}{}^{T}T_{e}+F\right)+T_{null}$$

where $J^{+}$ is the Moore-Penrose generalized inverse matrix of the matrix J, and $J^{+}=J^{T}{\left(JJ^{T}\right)}^{-1}$. $T_{null}=\lambda \cdot Null(J)$ is the general solution of cable tensions. λ is any positive real number. Null(J) is the null-space of the matrix $J^{T}$, and the cable tension can be effectively adjusted by setting the λ value.

In the actual operation of the BM-CDLR, it is necessary to calculate the determined cable tensions in real-time. Therefore, it is necessary to further research the optimization and distribution of the cable tension. The establishment of an optimization index model of the cable that meets the actual constraints of the system can uniquely determine the cable tensions and is also helpful to improve the safety performance of the BM-CDLR. The selected optimization model of the cable tension can be expressed as:

(7) $$\left\{ \begin{array}{l} \min f\\ s.t.J^{T}T-J_{e}{}^{T}T_{e}-F=0\\ \;\;T_{\min}-J^{+}\left(J_{e}{}^{T}T_{e}+F\right)\leq \lambda \cdot Null(J)\\ \;\;\lambda \cdot Null(J)\leq T_{\max}-J^{+}\left(J_{e}{}^{T}T_{e}+F\right) \end{array}\right.$$

where f indicates the optimized performance index. 

In the rehabilitation robot, the uniform distribution of the cable tensions is helpful to improve the ability of the rehabilitation device to resist and balance external interference. Therefore, the minimum variance of the cable tension is selected as the optimized performance index of the cable tension for the BM-CDLR. It can be written as:

(8) $$f(\lambda )=\frac{1}{4}\left(\sum_{i=1}^{4}{\left(t_{i}-t_{m}\right)}^{2}\right)$$

where $t_{m}$ is the mean value of cable tensions.

System stiffness is a major performance index of rehabilitation devices to evaluate the ability of the end-effector of the BM-CDLR to resist external interference. Therefore, the research of the system stiffness of the BM-CDLR has a positive effect on ensuring the safety of patients.

In the dynamic model of the BM-CDLR, the force generated by the elastic element is regarded as the external force acting on the traction point, and the magnitude and direction of this external force are required to be variable along the length of the cable. Therefore, the corresponding spinors of the cable and the elastic element should be simultaneously considered in the structure matrix when analyzing the system stiffness of the BM-CDLR. The system stiffness consists of two parts: active stiffness and passive stiffness. We can know from the mechanical balance Equation (3) that the elastic elements in the BM cable will increase the columns corresponding to the elastic elements in the structural matrix of the BM-CDLR (compared with the CDLR). In addition, the presence of elastic elements will also change the cable tensions, so the active stiffness will also be changed. The structural matrix of the system is related to the arrangement form of the cables, elastic elements, the position of the end-effector and other factors. In addition, the stiffness of the motion branch chain of the BM-CDLR system is also related to the cable tension. Therefore, the elastic elements in the BM cable will also change the passive stiffness of the system.

When calculating the system stiffness of the BM-CDLR, firstly, the cable tension can be calculated according to Equation (3). The elastic element can be regarded as a motion branch chain with a “special cable”; hence, Equation (3) can be further organized as:

(9) $$F=-J^{T}T+J_{e}{}^{T}T_{e}=J_{RO}{}^{T}T_{RO}$$

where $J_{RO}{}^{T}=\left[\begin{array}{cc} -J^{T} & J_{e}{}^{T} \end{array}\right]$, $T_{RO}={\left[\begin{array}{cc} T & T_{e} \end{array}\right]}^{T}$.

According to the definition of mechanism stiffness, the system stiffness of the BM-CDLR can be expressed as:

(10) $$K=\frac{dF}{dX}=\frac{dJ_{RO}{}^{T}}{dX}T_{RO}+J_{RO}{}^{T}\frac{dT_{RO}}{dX}=K_{S}+K_{T}$$

where X is the displacement of the traction point. $K_{S}$ is active stiffness. $K_{T}$ is passive stiffness.

We can know from Equation (10) that the active stiffness can be further expressed as:

(11) $$K_{S}=\frac{dJ_{RO}{}^{T}}{dX}T_{RO}=\frac{d\left(e_{1},\cdots ,e_{4},e_{e1},\cdots ,e_{e4},\right)}{dX}T_{RO}$$

where $e_{i}$ and $e_{ei}$ indicate the unit length vector of the cables and elastic elements, respectively. Since the elastic element is connected in parallel on the cable, they meet the condition $e_{i}=e_{ei}={\left[e_{xi}\;e_{yi}\;e_{zi}\;\right]}^{T}$.

Assume that when the lower limb traction point produces a slight displacement dX, the length vectors of the cables and the elastic elements change from $L_{i}$ and $L_{ei}$ to ${\bar{L}}_{i}$ and ${\bar{L}}_{ei}$, respectively, and their unit length vectors change from $e_{i}$ and $e_{ei}$ to ${\bar{e}}_{i}$ and ${\bar{e}}_{ei}$, respectively. At this time, the change of the unit length vector of the cables and the elastic elements can be expressed as:

(12) $$de_{i}=de_{ei}={\bar{e}}_{i}-e_{i}=\frac{{\bar{L}}_{i}}{\left\|{\bar{L}}_{i}\right\|}-\frac{L_{i}}{\left\|L_{i}\right\|}=\left[\begin{array}{ccc} e_{xi}{}^{2}-1 & e_{xi}e_{yi} & e_{xi}e_{zi}\\ e_{xi}e_{yi} & e_{yi}{}^{2}-1 & e_{yi}e_{zi}\\ e_{xi}e_{zi} & e_{yi}e_{zi} & e_{zi}{}^{2}-1 \end{array}\right]dX$$

We can know from Equation (10) that the passive stiffness can be further expressed as:

(13) $$K_{T}=J_{RO}{}^{T}\frac{dT_{RO}}{dX}=J_{RO}{}^{T}\frac{dT_{RO}}{dL_{i}}\frac{dL_{i}}{dX}=-J_{RO}{}^{T}\frac{dT_{RO}}{dL_{i}}J_{RO}{}^{T}$$

where:

(14) $$\frac{dT_{RO}}{dL_{i}}={\left[\frac{dt_{1}}{dL_{1}},\cdots ,\frac{dt_{4}}{dL_{4}},\frac{dt_{e1}}{dL_{e1}},\cdots ,\frac{dt_{e4}}{dL_{e4}}\right]}^{T}$$

where $dt_{i}/dL_{i}=k_{i}$ is the stiffness of the *i*th cable motion branch chain. According to the structure of the BM-CDU, $k_{i}$ is composed of cable stiffness and driving unit stiffness. However, they are connected in series in the system, and the stiffness of the cable is much smaller than the stiffness of the driving unit [25,36]. Hence, the stiffness of the *i*th motion branch chain can be expressed as:

(15) $$k_{i}=E_{i}A_{i}/L_{i0}$$

where $E_{i}$ is the elastic modulus of the cable material selected for the *i*th motion branch chain. $A_{i}$ is the cross-sectional area of the cable. $dt_{ei}/dL_{ei}=k_{ei}$ is the stiffness of the *i*th “special cable” (i.e., elastic element) motion branch chain.

Substitute Equations (11)–(14) into Equation (10), the system stiffness expression of the BM-CDLR can be obtained:

(16) $$K=K_{S}+K_{T}=-J_{RO}{}^{T}diag[k_{1},\cdots ,k_{4},k_{e1},\cdots ,k_{e4}]J_{RO}{}^{T}+H_{RO}T_{RO}$$

where $H_{RO}=dJ_{RO}{}^{T}/dX$.

In order to analyze the safety distribution rule of the BM-CDLR system, the singular value of the matrix ***K*** is selected to evaluate the system stiffness in this study. It can be expressed as:

(17) $$\sigma (K)=\sqrt{\lambda \left(K^{T}K\right)}$$

where $\lambda \left(K^{T}K\right)$ denotes the eigenvalues of the system stiffness matrix.

The minimum singular value *σ*_min_ can be expressed as:

(18) $$\sigma_{\min}=\min(\sigma (K))$$

where min(·) indicates the minimum function. Therefore, the larger the minimum singular value *σ*_min_ of the system stiffness matrix is, the larger the system stiffness of the BM-CDLR is.

### 2.3. Safety Evaluation of the BM-CDLR

Safety is one of the important performance indicators of rehabilitation medical devices. In CDLR, because the cable can only provide a positive tension force, it is difficult to ensure the safety of patients and realize the real-time control of the system. During the operation of the system, the cable tension and the system stiffness are evenly distributed in the central area of the CDLR workspace. However, the cable tension distribution in the boundary area of the workspace is seriously uneven, and there is even the pseudo-drag phenomenon, which leads to the reverse increase of system stiffness in the boundary area of the workspace [25]. Therefore, the position, cable tension, and system stiffness have a great impact on the safety of the BM-CDLR system. When the cables of the CDLR are replaced by the BM cables, there are no relevant research reports about the effect of the elastic element in the BM cable on the cable tension distribution, system stiffness distribution, and the safety of the BM-CDLR. Therefore, it is necessary to further study the change rule of the safety for the BM-CDLR system and the influence of related parameters on the safety of the BM-CDLR system.

The movement ability of patients with different degrees of lower limb movement disorders is different. The effect of the movement speed of the lower limb on safety of the BM-CDLR should be considered in the safety evaluation of the system to ensure the safety of patients in the rehabilitation training. Therefore, the position, the tension of the BM cable, the system stiffness, and the movement speed of the lower limb should be considered in the safety evaluation of the robot. Then, the safety performance factors and the speed influence function will be defined to study the evaluation method and the distribution rule of safety of the BM-CDLR.

As shown in Figure 1, *P* indicates any position of the traction point in the workspace. *Q* and *M* indicate the center points of the horizontal section where the traction point is located at any position and the upper surface of the workspace, respectively. The angle between the line that is the pulley corresponding to the movement branch chain with the minimum tension force of the BM cable and the traction point *P* and the horizontal plane is *θ*. *θ_P_*, *θ_Q_*, and *θ_M_* indicate the angle *θ* when the traction point is at the *P*, *Q*, and *M* positions respectively.

The position performance factors ${℘}_{Ph}$ and ${℘}_{Pv}$ are respectively defined to measure the distance from the traction point *P* to the vertical midline and the upper surface of the BM-CDLR workspace. They can be expressed as:

(19) $$\left\{ \begin{array}{l} {℘}_{Ph}=\tan\theta_{P}/\tan\theta_{Q}\\ {℘}_{Pv}=\tan\theta_{M}/\tan\theta_{Q} \end{array}\right.$$

Assume that the position of the pulley corresponding to the movement branch chain with the minimum tension force of the BM cable is [$\begin{array}{ccc} x_{bi}{}^{*} & y_{bi}{}^{*} & z_{bi}{}^{*} \end{array}$], there is $\tan\theta =\left(z_{bi}{}^{*}-z\right)/\sqrt{{\left(x_{bi}{}^{*}-x\right)}^{2}+{\left(y_{bi}{}^{*}-y\right)}^{2}}$.

The BM cable tension is the direct factor to achieve the desired movement and balance the external interference force. When the constraint force of the traction point is larger in all directions and the distribution of the constraint force is more uniform, it is the more difficult to change the motion state of the lower limbs. Therefore, the BM cable tension force performance factors ${℘}_{TP}$ and ${℘}_{T}$ are defined to measure the uniformity of the distribution of the constraint forces of the BM cable on the lower limb at the current position and the distribution of the minimum tension force of the BM cable in the workspace. They can be expressed as:

(20) $$\left\{ \begin{array}{l} {℘}_{TP}=T_{\min}^{P}/T_{\max}^{P}\\ {℘}_{T}=T_{\min}^{P}/T_{\max}^{\min} \end{array}\right.$$

where $T_{\min}^{P}$ and $T_{\max}^{P}$ respectively indicate minimum value and maximum value of the tension force of the BM cable at the current position. $T_{\max}^{\min}$ indicates maximum value of minimum tension forces of the BM cable in the workspace. The tension force of the BM cable can be calculated using Equation (6):

(21) $$\left\{ \begin{array}{l} T_{\min}^{P}=\min(T)\\ T_{\max}^{P}=\max(T) \end{array}\right.$$

System stiffness has great significance to improve the safety of the BM-CDLR. Therefore, the system stiffness performance factors ${℘}_{KP}$ and ${℘}_{K}$ are defined to evaluate the distribution uniformity of system stiffness in any position and workspace:

(22) $$\left\{ \begin{array}{l} {℘}_{KP}=\sigma_{P\min}/\sigma_{P\max}\\ {℘}_{K}=\sigma_{P\min}/\sigma_{\max}^{\min} \end{array}\right.$$

where $\sigma_{P\min}$ and $\sigma_{P\max}$ respectively represent minimum singular value and maximum singular value of the matrix ***K*** at the current position. $\sigma_{\max}^{\min}$ represents maximum value of minimum singular value of the system stiffness matrix in the workspace.

Combined with mechanical analysis of the BM-CDLR and Equations (19)–(22), it can be seen that when the larger the performance factor ${℘}_{Ph}$ is, the smaller the distance from the point *P* to the line *QM* is, the more uniform the distributions of the BM cable tension and the system stiffness are, which means that the distribution of the ${℘}_{TP}$ and ${℘}_{KP}$ are more uniform, and the better the ability of the robot to resist external interference is, which means that the safety of the BM-CDLR will be better. Similarly, the larger the performance factor ${℘}_{Pv}$ is, the smaller the distance from the point *P* to the upper surface of the workspace is, the larger the BM cable tensions and the system stiffness are, which means that the distribution of the ${℘}_{T}$ and ${℘}_{K}$ are more concentrated, and the ability to resist external interference and the safety of the BM-CDLR will be better. Therefore, the larger the value of the above performance factors are, the better the safety of the BM-CDLR will be.

It can be seen from the above discussion that the tension force of the BM cable is related to the position of the traction point and the structure of the BM-CDLR. The system stiffness of the BM-CDLR is related to the tension force of the BM cable, the position of the traction point, and the structure of the BM-CDLR. Therefore, there is a close relationship among the safety performance factors.

#### 2.3.1. Structural Safety

The structural safety of the BM-CDLR is closely related to the position of the traction point, the tension force of the BM cable, and the system stiffness of the BM-CDLR. Therefore, based on the above safety performance factors, the structural safety evaluation index *S_struc_* of the BM-CDLR system is given using the weighted method in this study. The size of the structural safety evaluation index *S_struc_* can be used to measure the degree of the structural safety of the BM-CDLR system. The structural safety evaluation index *S_struc_* of the BM-CDLR can be expressed as:

(23) $$S_{struc}=\left(\eta_{TP}{℘}_{TP}+\eta_{KP}{℘}_{KP}\right)\cdot \left(\eta_{T}{℘}_{T}+\eta_{K}{℘}_{K}\right)\cdot \left(\eta_{Pv}{℘}_{Pv}+\eta_{Ph}{℘}_{Ph}\right)$$

where $\eta_{TP}$, $\eta_{KP}$, $\eta_{T}$, $\eta_{K}$, $\eta_{Pv}$, and $\eta_{Ph}$ represent the weight coefficients of the corresponding safety performance factors, respectively, and they meet the following condition:

(24) $$\left\{ \begin{array}{l} \eta_{TP}+\eta_{KP}=1\\ \eta_{T}+\eta_{K}=1\\ \eta_{Pv}+\eta_{Ph}=1 \end{array}\right.$$

#### 2.3.2. Use Safety

The use safety of the BM-CDLR refers to the safety of rehabilitation equipment for the patient in the actual rehabilitation training. In the actual rehabilitation training process, different patients have different movement disorders and training needs, and different patients have different abilities to withstand movement intensity. Therefore, the influence of the movement speed of the traction point on the safety of the patient should be considered to evaluate the use safety of the BM-CDLR based on the basis of the structural safety evaluation index. The size of the use safety evaluation index *S_use_* can be used to measure the level of the use safety of the BM-CDLR system. It can be expressed as:

(25) $$S_{use}=S_{struc}\cdot f\left(v\right)$$

where *f*(*v*) indicates the speed influence function. It can be used to measure the influence degree of the movement speed of the traction point on the safety of the BM-CDLR. Therefore, the speed influence function *f*(*v*) must meet the following conditions:

(1) The larger the motion speed of the traction point is, the poorer the safety of the BM-CDLR is.

(2) Assuming that the movement speed of the traction point *P* is $v_{P}=\left\|\dot{X}\right\|$, when the movement speed of the traction point *P* is $v_{P}$ = 0, that is, the lower limbs are in a static state, the influence of the movement speed on the safety of the BM-CDLR is not considered, namely, $S_{use}=S_{struc}$.

(3) Assuming that the maximum allowable safe speed determined by the patient’s motion tolerance ability is $v_{\max}$, when the movement speed of the traction point *P* is $v_{P}$ = $v_{\max}$, the movement speed of the traction point *P* reaches the maximum value, and we define the use safety index of the BM-CDLR *S_use_* = 0 at this time, and *f*(*v*) = 0.

According to the above conditions, the speed influence function shown in Equation (26) can be selected as follows in this study:

(26) $$f\left(v\right)=1-v_{P}/v_{\max}$$

The speed influence function shown in Equation (26) obviously satisfies Conditions (1)–(3), and its proof is easy [25]; therefore, this paper will not prove them. In addition, the maximum allowable safe speed that the patient can withstand should be determined by combining with the patient’s lower limb movement disorder level and the professional opinions from the rehabilitation physician. It is worth noting that the value range of the use safety index of the BM-CDLR is *S_use_* ∈ [0, *S_struc_*].

Finally, the weight coefficients are determined by combining with the importance and contribution of each safety performance factor to the safety of the BM-CDLR system when calculating the safety evaluation index of the BM-CDLR. In order to analyze the influence of the elastic elements in the BM cable on the safety of the BM-CDLR, this study selects the weight coefficients in Ref. [25] to facilitate a comparative analysis. Hence, the weight coefficients selected in this study are $\eta_{TP}$ = 0.6, $\eta_{KP}$ = 0.4, $\eta_{T}$ = 0.55, $\eta_{K}$ = 0.45, $\eta_{Pv}$ = 0.6, and $\eta_{Ph}$ = 0.4.

## 3. Results and Discussion

### 3.1. Distribution Analysis of the Structural Safety of the BM-CDLR

In order to analyze the influence of the elastic elements in the BM cable on the safety of the BM-CDLR, this study will take the specific BM-CDLR with the following structural parameters as an example for discussion: the positions of the pulleys are ***b***_1_ = [500, 0, 800]^T^ mm, ***b***_2_ = [0, 0, 800]^T^ mm, ***b***_3_ = [0, 1680, 800]^T^ mm, and ***b***_4_ = [500, 1680, 800]^T^ mm. The allowable range of the cable tension is ***T*** ∈ [10, 500] N. The force generated by the lower limb acting on the traction point is regarded as a constant force in the vertical direction to facilitate experimental verification, that is ***F*** = [0, 0, −98] N. The adopted cables of the BM-CDLR are steel cables, the minimum breaking force of which is 1.69 × 10^3^ N, the elastic modulus *E_i_* = 1.9402 × 10^5^ MPa, and the diameter is 1.8 mm in the standard GB/T 20118-2006. 6 × 7 + IWS (Iron Wire Steel). 

We can be known that $T_{\max}^{\min}$ = 419.112 N, $\sigma_{\max}^{\min}$ = 5.8072 × 10^5^ by calculation. In this study, the BM cables composed of three groups of elastic elements are selected to study the influence of the elastic elements on the safety of the BM-CDLR. The parameters of the elastic elements are shown in Table 1. In addition, the material of the elastic elements in the BM cable is 304 stainless steel, so the damping coefficient of the elastic elements is $c_{ei}$ = 0.001 N·s/m. Therefore, the effects of the stiffness coefficient and the free length of the elastic elements in the BM cable on the safety of the BM-CDLR are mainly studied here.

**Figure 2:**
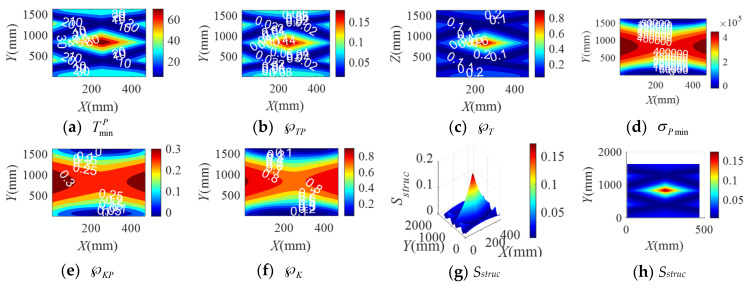
The distribution of safety performance factors and structural safety evaluation index on the horizontal section Z = 0.5 m (Group A elastic elements).

**Figure 3:**
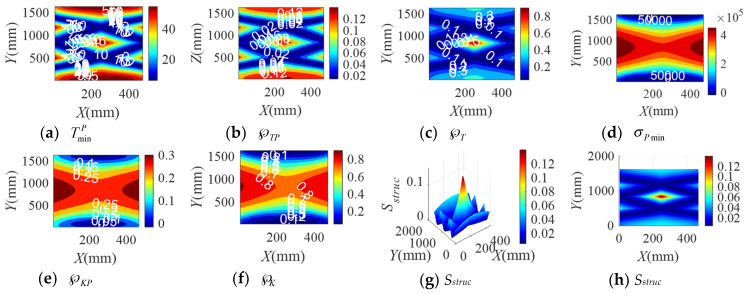
The distribution of safety performance factors and structural safety evaluation index on the horizontal section Z = 0.5 m (Group B elastic elements).

**Figure 4:**
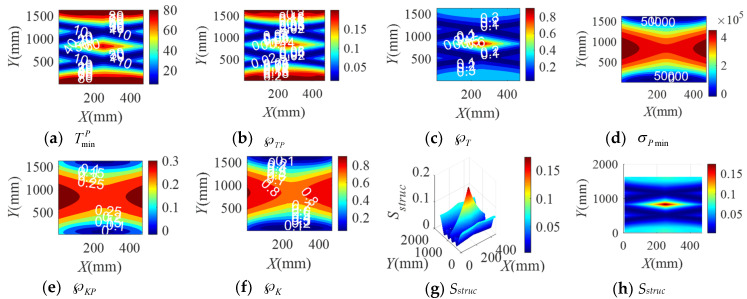
The distribution of safety performance factors and structural safety evaluation index on the horizontal section Z = 0.5 m (Group C elastic elements).

**Figure 5:**
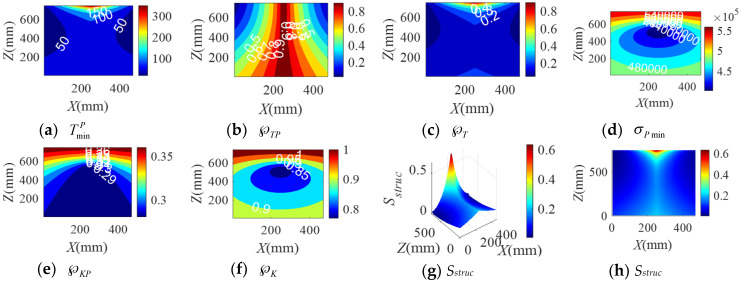
The distribution of safety performance factors and structural safety evaluation index on the vertical section Y = 0.84 m (Group A elastic elements).

**Figure 6:**
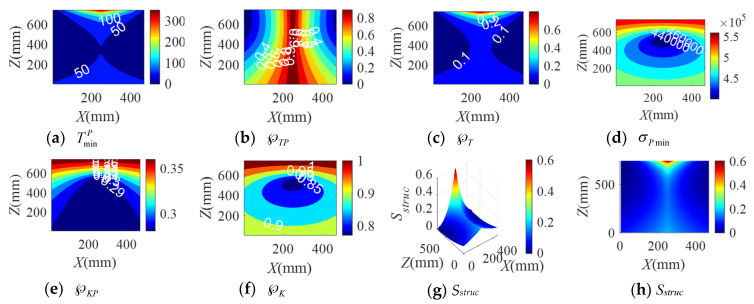
The distribution of safety performance factors and structural safety evaluation index on the vertical section Y = 0.84 m (Group B elastic elements).

**Figure 7:**
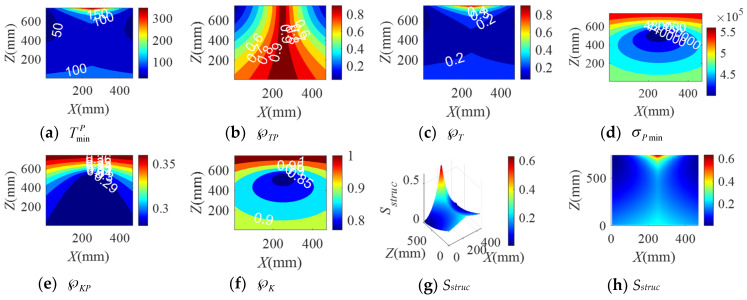
The distribution of safety performance factors and structural safety evaluation index on the vertical section Y = 0.84 m (Group C elastic elements).

When the BM-CDLR respectively adopts the BM cables with the group A, B, and C elastic elements, the distribution rules of the safety performance factors and the structural safety evaluation index of the BM-CDLR in the horizontal section Z = 500 mm are shown in Figure 2, Figure 3 and Figure 4, respectively. Their distribution rules in the vertical section Y = 840 mm are shown in Figure 5, Figure 6 and Figure 7, respectively. The related calculation results are shown in Table 2 and Table 3, in which the CDLR indicates the calculation results of the CDLR in [25].

It can be seen from Figure 2, Figure 3, Figure 4, Figure 5, Figure 6 and Figure 7 that the distribution maps of $\sigma_{P\min}$, ${℘}_{KP}$, and ${℘}_{K}$ of the BM-CDLR are basically consistent with the research results in [25]. According to Equations (11), (13) and (16), it can be seen that the stiffness and force changes of the elastic elements are introduced in the calculation process of the system stiffness of the BM-CDLR. However, the elastic elements and the selected steel cables are connected in parallel, and the magnitude of the stiffness values is quite different; hence, the elastic elements in the BM cable have little effect on the system stiffness and stiffness performance factors of the BM-CDLR. The distributions of the safety performance factors and structural safety evaluation index are symmetrical about the center position of the section. $T_{\min}^{P}$, ${℘}_{TP}$, ${℘}_{T}$, and *S_struc_* gradually decrease from the center position of the horizontal section of the boundary area of the horizontal section. These basic distribution rules are consistent with the distribution rules of the CDLR in [25].

It can be seen from Figure 2a–c, Figure 3a–c and Figure 4a–c that the distribution maps of $T_{\min}^{P}$, ${℘}_{TP}$, and ${℘}_{T}$ of the BM-CDLR show a parallelogram distribution, and there is a reverse growth in the boundary area of the horizontal section of the BM-CDLR workspace. We can know from Equations (3) and (4) that this phenomenon is caused by the change of the difference between the actual length and the free length of the elastic element, which can solve the pseudo-drag phenomenon of the CDLR in the boundary area. The results of the literature [25] show that the distribution map of $T_{\min}^{P}$, ${℘}_{TP}$, and ${℘}_{T}$ of the CDLR is an approximate hexagonal distribution, and there is a distortion of less than zero in the boundary area of the horizontal section of the workspace, which has a big negative impact to the safety of patients. It shows that the BM cables, instead of the ordinary cable, can improve the tension force performance factors and the safety of the BM-CDLR system.

When the free length of the elastic element in the BM cable increases from 0.9 m to 1 m, there is a more obvious reverse growth at the boundary of the horizontal section Z = 0.5 m, compared with the results shown in Figure 2 and Figure 3; the area of the central area with $T_{\min}^{P}$ ≥ 10 N, ${℘}_{TP}$ ≥ 0.1, and ${℘}_{T}$ ≥ 0.02 is reduced. The values $T_{\min}^{P}$, ${℘}_{TP}$, and ${℘}_{T}$ will be significantly reduced. This is detrimental to the safety of the BM-CDLR. The distribution law of *S_struc_* on the horizontal section Z = 0.5 m is basically unchanged, but the reverse growth of *S_struc_* in the boundary area is relatively obvious. The area with *S_struc_* ≥ 0.02 in the central area of the horizontal section is reduced by approximately 44.82%. When the free length of the elastic element is larger than 0.9 m, in the section Z = 500 mm, the value *S_struc_* is significantly reduced in the central area of the horizontal section, which means that the safety of the BM-CDLR system is also decreased. Compared with the results shown in Figure 5 and Figure 6, the areas where the reverse growth of $T_{\min}^{P}$ and ${℘}_{T}$ account for about 50% of the workspace in vertical section Y = 0.84 m, the reverse growth being especially more obvious between 300 mm ≤ Z ≤ 400 mm, which can solve the pseudo-drag phenomenon at the boundary area and increase the constraint forces on the traction point. Moreover, the value ${℘}_{TP}$ is reduced by about 0.1 in the central area of the vertical section Y = 840 mm. The reduction of ${℘}_{TP}$ at the boundary area has little effect on the lower limb rehabilitation training tasks, because the lower limb rehabilitation training tasks should be generally planned in the middle area of the workspace [25,26]. The distribution rules of the structural safety evaluation index *S_struc_* are basically the same. The structural safety evaluation index *S_struc_* in the central area has a certain increase, but it is not obvious. It indicates that when the free length of the elastic element is larger than 0.9 m, in the vertical section Y = 840 mm, the *S_struc_* in the central area of the vertical section will be increased, which can improve the safety of the system. 

When the stiffness coefficient of the elastic element in the BM cable increases from 200 N/m to 300 N/m, compared with the results shown in Figure 2 and Figure 4, the reverse growth of $T_{\min}^{P}$, ${℘}_{TP}$, and ${℘}_{T}$ in the boundary area is more obvious, which is caused by the change of the free length and the stiffness coefficient of the elastic element. The area of the central area with $T_{\min}^{P}$ ≥ 10 N, ${℘}_{TP}$ ≥ 0.1 and ${℘}_{T}$ ≥ 0.02 is reduced. Their maximum values are obviously increased. This is helpful to improve the safety of the BM-CDLR. The distribution law of *S_struc_* is basically unchanged, but there is a relatively obvious reverse growth phenomenon at the boundary. The area with *S_struc_* ≥ 0.02 in the central area of the horizontal section is reduced. When the stiffness coefficient of the elastic element is larger than 200 N/m, in the horizontal section Z = 500 mm, the value *S_struc_* for the safety of the BM-CDLR system is significantly increased in the central area of the horizontal section. Compared with the results shown in Figure 5 and Figure 7, the values of $T_{\min}^{P}$ and ${℘}_{T}$ are obviously increased in the vertical section Y = 840 mm, and the reverse growth of $T_{\min}^{P}$ and ${℘}_{T}$ are obviously increased at the central and lower areas. Moreover, the value ${℘}_{TP}$ is also increased in the central and boundary areas. The structural safety evaluation index *S_struc_* in the lower area is obviously increased. When the stiffness coefficient of the elastic element is larger than 200 N/m, in the vertical section Y = 840 mm, the value *S_struc_* in the central and lower areas of vertical section is increased, which can improve the safety of the system. 

It is worth noting that when the free length and the stiffness coefficient of the elastic element in the BM cable are 1 m and 200 N/m, respectively, in order to obtain the better safety performance of the BM-CDLR, the training task should be planned in the Z ∈ [300, 400] mm area.

In summary, when the free length and stiffness coefficient of the elastic element in the BM cable are changed, the change of the safety performance factors $T_{\min}^{P}$, ${℘}_{TP}$, and ${℘}_{T}$ the structural safety evaluation index *S_struc_* are obvious. The changes of the safety performance factors $\sigma_{P\min}$, ${℘}_{KP}$, and ${℘}_{K}$ are not obvious. In addition, when the lower limb rehabilitation training tasks are performed in different workspace areas, the free lengths of the selected elastic elements in the BM cable are different, and the stiffness coefficient of the elastic elements in the BM cable should be appropriately increased to obtain the best safety of the BM-CDLR. 

### 3.2. Experimental Study of the Use Safety of the BM-CDLR

**Figure 8:**
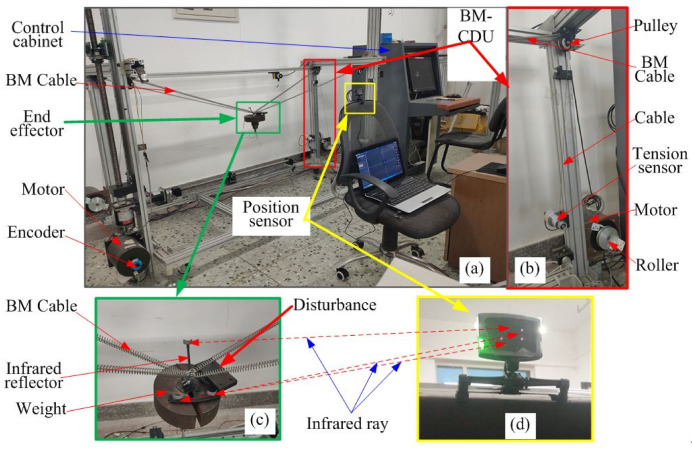
The experiment system of the BM-CDLR. (**a**) Experiment system; (**b**) cable-driven unit (CDU); (**c**) end-effector; (**d**) position sensor.

An experimental system platform with four BM cables was established to analyze and verify the safety evaluation method of the BM-CDLR system, as shown in Figure 8a. It consists of four BM-CDUs, which include four torque motors, four photoelectric encoders, four tension sensors and pulleys, and a position sensor. The BM-CDU is shown in Figure 8b. A disturbance with a mass of 1.2 kg is fixed on the edge of the end-effector with a mass of 10 kg. The position data of the end-effector are collected by the position sensor. The BM cable tension data are collected by the tension sensors. The elastic elements are connected in parallel on the steel cable between the end-effector and the pulley to form BM cables. In this study, the maximum allowable movement speed is $v_{\max}=0.75\;m/s$.

In order to verify the use safety evaluation method of the BM-CDLR and the influence rules of the elastic elements in the BM cable on the safety of the BM-CDLR, the same motion trajectory as in [25] is selected as the desired trajectory of this study to facilitate the comparative analysis of the experimental results:

(27) $$\left\{ \begin{array}{l} x=x_{0}+10\left(t-t_{0}\right)\\ y=y_{0}\\ z=z_{0} \end{array}\right.\;(0\leq t\;\leq 5\;s,\;\mathrm{and}\;25\;s\leq t\;\leq 30\;s\;)$$

(28) $$\left\{ \begin{array}{l} x=Ri\cdot \cos((t-t_{0})\pi /10)+x_{0}\\ y=Ri\cdot \sin((t-t_{0})\pi /10)+y_{0}\\ z=z_{0} \end{array}\right.\;(5\;s\;<\;t\;\leq 25\;s,\;\mathrm{and}\;30\;s\;<\;t\;\leq 50\;s)$$

We can know from Equations (27) and (28) that the desired motion trajectory are straight lines when *t* ∈ [0, 5] s and *t* ∈ (25, 30] s, and circles with a center of ($x_{0}$, $y_{0}$, $z_{0}$) and a radius of *Ri* when *t* ∈ [25, 30] s and *t* ∈ (30, 50] s. Let ($x_{0}$, $y_{0}$, $z_{0}$) = (250, 840, 500) mm, *R*1 = 50 mm, and *R*2 = 100 mm. Hence, the speeds of the end-effector are v = 10 mm/s (*t* ∈ [0, 5] s), v ≈ 15.71 mm/s (*t* ∈ (5, 25] s), v = 10 mm/s (*t* ∈ (25, 30] s), and v ≈ 31.42 mm/s (*t* ∈ (30, 50] s).

The use safety evaluation index of the BM-CDLR satisfies the condition *S_use_* ∈ [0, *S_struc_*]. According to the research results of the structural safety of the BM-CDLR in Section 4, it can be seen that the value *S_struc_* is relatively small in the horizontal section Z = 500 mm. In addition, in the initial stage of the rehabilitation training, the motion speed of the lower limbs is not allowed to be too large, which will lead to a smaller and more concentrated use safety evaluation index *S_use_* of the BM-CDLR. Therefore, in the actual application process, it is necessary to combine the safety performance factors and the value *S_use_* for comprehensively evaluating the use safety of the BM-CDLR. In this experiment, the data value that can directly reflect the safety of the BM-CDLR and patients is the size of the motion error of the end-effector. The larger the motion error value of the end-effector is, the poorer the use safety of the BM-CDLR is. Therefore, under the influence of external interference, this study will combine the tracking error of the motion trajectory, the safety performance factors, and the safety evaluation index to prove the rationality of the safety evaluation method of the BM-CDLR.

**Figure 9:**
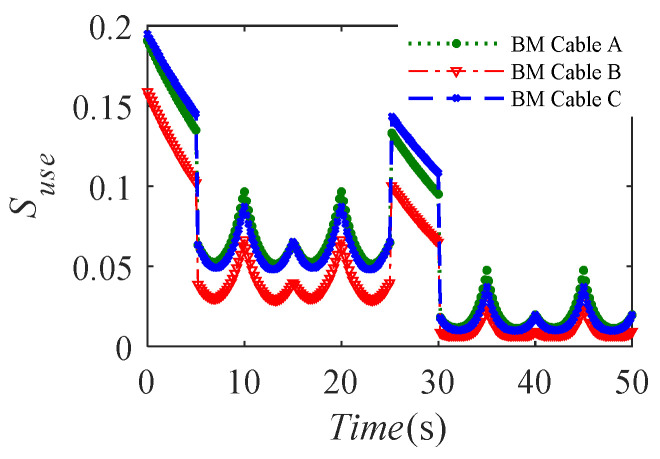
The use safety evaluation index *S_use_* of the BM-CDLR.

The change curves of the use safety evaluation index *S_use_* of the lower limb rehabilitation robot system driven by the BM cables with the elastic elements in Table 1 are shown in Figure 9. It can be seen that the use safety evaluation index *S_use_* has certain periodic changes on the circular trajectories with *R*1 and *R*2, which is mainly caused by the position performance factors. The horizontal section of the BM-CDLR workspace is a rectangle. The relative distance between the circular trajectory and the rectangular boundary has a certain periodicity change. In addition, the sudden change of the desired motion trajectory will lead to the sudden change of the tension force of the BM cables. From the definition Equation (10) of the system stiffness, it can be seen that the system stiffness of the BM-CDLR will also be changed suddenly, but it is not obvious. Therefore, the sudden changes in the tension force of the BM cable and the system stiffness lead to a sudden change of the use safety evaluation index *S_use_* at *t* = 5 s, 25 s, and 30 s. It is worth noting that the distance from the end-effector to the line *QM* is smaller in the *X*-axis direction than in the *Y*-axis direction at *t* = 10 s, 20 s, 35 s, and 45 s, therefore, the safety performance factors ${℘}_{Ph}$, ${℘}_{Pv}$, ${℘}_{TP}$, and ${℘}_{T}$ are larger, and the increased value of *S_use_* is larger at *t* = 10 s, 20 s, 35 s, and 45 s than at *t* = 15 s and 40 s.

In addition, the movement speed of the end-effector on a circular trajectory with *R*2 is greater than that on a circular trajectory with *R*1, and the distance between the end-effector and the circular trajectory with *R*2 is greater than the distance between the end-effector and the circular trajectory with the radius *R*1, so the minimum values of the BM cable tensions and system stiffness are smaller on the circular trajectory with *R*2 than on the circular trajectory with *R*1, while the distribution of the cable tensions and system stiffness on the circular trajectory with *R*2 are more uneven. Hence, the use safety of the BM-CDLR is better between 5 and 25 s than between 30 and 50 s.

By comparing the use safety evaluation index *S_use_* of the lower limb rehabilitation robot driven by the BM cable of Groups A and B, one can know that the use safety evaluation index *S_use_* is significantly reduced in the horizontal section of Z = 500 mm when the free length of the elastic element in the BM cable increases from 0.9 m to 1 m. The reduction of the index *S_use_* is more obvious in the central area. By comparing the use safety evaluation index *S_use_* of the lower limb rehabilitation robot driven by the BM cable of Groups A and C, one can know that the use safety evaluation index *S_use_* is increased on the centerline of the horizontal section Z = 500 mm (*t* ∈ [0, 5] s and *t* ∈ [25, 30] s in Figure 9) when the stiffness coefficient of the elastic element in the BM cable increases from 200 N/m to 300 N/m. The use safety evaluation index *S_use_* is decreased on the circular trajectory with the radii *R*1 and *R*2, but the reduced value is not obvious. In addition, the change of the use safety evaluation index *S_use_* is not a simple translation when the free length and the stiffness coefficient of the elastic elements in the BM cable are changed.

**Figure 10:**
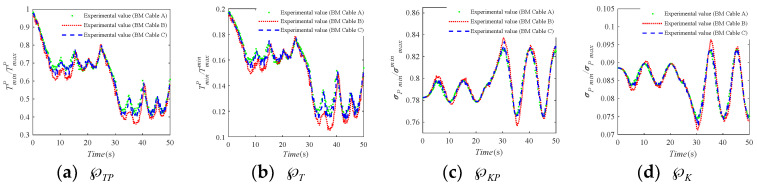
The safety performance factors of the BM-CDLR.

The three groups of the elastic elements in Table 1 are used in the BM cable when the test experiments are performed on the BM-CDLR experimental system, and the corresponding test experiments are denoted as Experiment A, Experiment B, and Experiment C, respectively. The relative data detected by the tension sensors and the position sensor are transmitted back to the upper computer, and the real-time change curves of the safety performance factors calculated by combining Equations (6), (10), (20) and (23) are shown in Figure 10. It can be seen that the trend of the safety performance factors is consistent with the three groups of test experiments. Comparing the calculation results of Experiment A and Experiment B, it can be seen that the safety performance factors ${℘}_{TP}$ and ${℘}_{T}$ are decreased in the horizontal section of Z = 500 mm, when the stiffness coefficient of the elastic element in the BM cable is 200 N/m and the free length of the elastic element in the BM cable increases from 0.9 m to 1 m, and the reduction amplitudes on the large circle with the radius *R*2 are larger than those on the small circle with the radius *R*1. However, the change of the safety performance factors ${℘}_{KP}$ and ${℘}_{K}$ is not obvious. Comparing the calculation results of Experiment A and Experiment C, it can be seen that the change of the safety performance factors ${℘}_{TP}$ and ${℘}_{T}$ is decreased, but not obvious in the horizontal section of Z = 500 mm, when the stiffness coefficient of the elastic element in the BM cable is 200 N/m and the free length of the elastic element in the BM cable is increased from 0.9 m to 1 m. The reduction amplitudes on the large circles with the radius *R*2 and radius *R*1 are almost indistinguishable. At the same, the change of the safety performance factors ${℘}_{KP}$ and ${℘}_{K}$ is not obvious in Experiment A and Experiment C. It further shows that the change of the stiffness coefficient and free length of the elastic element in the BM cable has little effect on the system stiffness of the BM-CDLR.

**Figure 11:**
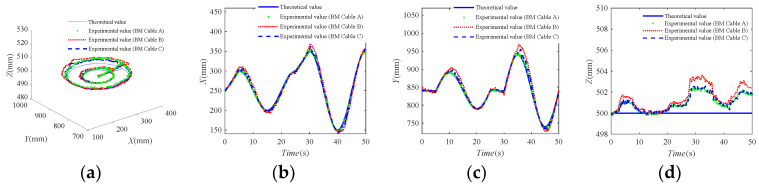
The tracking motion trajectories of the BM-CDLR. (**a**) Tracking trajectories; (**b**) tracking trajectories on the *X*-axis. (**a**) Tracking trajectories; (**b**) Tracking trajectories on the *X*-axis; (**c**) tracking trajectories on the *X*-axis; (**d**) tracking trajectories on the *X*-axis.

In the three groups of test experiments, the motion tracking trajectories of the end-effector with the action of external interference are shown in Figure 11. It can be seen that the motion error between the test experimental trajectories and the desired motion trajectory of the end-effector is small, which can meet the needs of the general functional training. It shows that the BM-CDLR experimental system has a high motion tracking ability.

The errors of the tracking trajectories on the small circle and large circle are shown in Table 4. The error value between the theoretical value and the experimental value is significantly smaller on the circle with *R*1 than that on the circle with *R*2. Combined with the definition equations of the safety performance factors (19), (20), and (23) and the definition Equation (26) of the use safety of the robot, one can know that ${℘}_{Ph}$, ${℘}_{Pv}$, ${℘}_{TP}$, and ${℘}_{T}$, ${℘}_{KP}$, and ${℘}_{K}$ are larger on the circle with *R*1 than on the circle with *R*2, and the movement speed of the end-effector is smaller on the circle with *R*1 than on that on the circle with *R*2; therefore, the ability of the BM-CDLR to resist external interference is stronger on the circle with *R*1, which means that the error value between the theoretical value and the experimental value should be smaller on the circle with *R*1. This point is consistent with the experimental results. The experimental results indicate that the use safety evaluation method and index are reasonable and can be used to evaluate the use safety of the BM-CDLR.

By comparing with the experimental results of Test Experiment A and Test Experiment B, one can know that the motion tracking error of the end-effector increases obviously in the horizontal section Z = 500 mm when the stiffness coefficient of the elastic element in the BM cable is 200 N/m and the free length of the elastic element increases from 0.9 m to 1 m. This will reduce the use safety of the BM-CDLR system. By comparing with the experimental results of test experiment A and Test Experiment C, one can know that the motion tracking error of the end-effector is increased in the horizontal section Z = 500 mm when the free length of the elastic element is 0.9 m, and the stiffness coefficient of the elastic element in the BM cable increases from 200 N/m to 300 N/m; however, it is not obvious. It will increase the use safety of the BM-CDLR, but the effect of the free length of the elastic element on the experimental results is not obvious. The test results are consistent with the change rules in the use safety evaluation index *S_use_* and safety performance factors of the BM-CDLR system shown in Figure 9 and Figure 10.

Compared with the test results of the CDLR driven by an ordinary cable in [25], when the BM cable is used to drive the motion of the end-effector, the motion errors of the end-effector are significantly reduced, especially in Test Experiment A and Test Experiment C. It shows that the introduction of the BM cable can not only increase the use safety of the BM-CDLR, but also improve the configuration problem of the active stiffness and the passive stiffness for the BM-CDLR to further optimize the human-machine interaction compliance of the BM-CDLR in the future research. 

Through the test experiments and simulation results of the safety of the BM-CDLR, it can be seen that the introduction of the BM cable into the CDLR can effectively improve the motion performance and safety of the rehabilitation robot. However, the appropriate structural parameters of the elastic element in the BM cable should be selected in different workspace areas to improve the rehabilitation training effect and the use safety performance of the BM-CDLR to the greatest extent. For example, when the stiffness coefficient of the elastic element in the BM cable is 200 N/m and the free length is 1 m, the reasonable area of the end-effector should be the central area and Z ∈ [300, 400] mm of the BM-CDLR workspace. When the end-effector is working at the section of Z = 500 mm, it is more reasonable to select the elastic element with the stiffness coefficient 200–250 N/m and the free length 0.9 m to construct a BM cable. However, if the selected structural parameters of the elastic elements in the BM cable are unreasonable, the working performance and safety performance of the BM-CDLR may be reduced.

## 4. Conclusions

A mechanical model of the BM cable is presented based on the Hill muscle model to construct the BM-CDLR in this paper. The safety evaluation index of the BM-CDLR system is given. The rationality of the proposed safety evaluation index of the BM-CDLR is verified by test experiments. The following conclusions are obtained through numerical simulation and test experimental results:

(1) The introduction of the BM cable can solve the pseudo-drag problem of the CDPR.
(2) The safety of the BM-CDLR is better in the middle and upper areas of the workspace than that of the boundary area. The smaller movement speed of the lower limb is helpful to improve the safety of the BM-CDLR. However, in order to enhance the training effect of the BM-CDLR, it is necessary to determine a feasible movement speed of the lower limbs in actual training according to the patient’s movement ability and professional evaluation given by the rehabilitation physiotherapist.
(3) The stiffness coefficient and the free length of the elastic elements in the BM cable have a greater impact on the motion performance and safety of the BM-CDLR system. Therefore, it is necessary to choose the stiffness coefficient and the free length of the elastic elements in the BM cable according to the planned training task, the safety performance factors, and the safety evaluation index in practical applications.

In the future work, we will further study the selection method of the structural parameters of the elastic elements in the BM cable in different workspace areas based on the safety distribution rules of this study. Meanwhile, the compliance control strategy and human-machine interaction experiment of the BM-CDLR need to be further studied.

## Figures and Tables

**Table 1:** Parameters of the elastic element in the BM cable.

Group No.	*k_ei_* (N/m)	*L_i_*_0_ (m)	*P*_0_ (mm)	*d* × *D*_0_ (mm × mm)
**A**	200	0.9	4.7	1.4 × 11
**B**	200	1.0	5.3	1.4 × 11
**C**	300	0.9	7.0	1.4 × 11

Note: *P*_0_ indicates the pitch of the elastic element. *d* indicates the wire diameter of the elastic element. *D*_0_ indicates the outer diameter of the elastic element.

**Table 2:** Calculation results with different elastic elements in the BM cables in horizontal section Z = 500 mm.

Group No.	Compared with A, Area Change (%)				Maximum Values						
	$T_{\min}^{P}\geq 10N$	${℘}_{TP}\geq 0.1$	${℘}_{T}\geq 0.02$	*S_struc_* ≥ 0.02	$T_{\min}^{P}$	${℘}_{TP}$	${℘}_{T}$	$\sigma_{P\min}/\times 10^{5}$	${℘}_{KP}$	${℘}_{K}$	*S_struc_*
**A**	/			/	78.6132	0.9337	0.1876	5.8092	0.9086	0.3865	0.1706
**B**	reduce 44.8			reduce 44.8	58.8519	0.9131	0.1140	5.8092	0.9131	0.3865	0.1378
**C**	reduce 14.1			reduce 13.6	89.5437	0.9180	0.2137	5.8111	0.9085	0.3866	0.1707
**CDLR**	/				70.31	0.9051	0.1651	5.8053	0.9113	0.3826	1.504

**Table 3:** Calculation results with different elastic elements in the BM cables in vertical section Y = 840 mm.

Group No.	Maximum Values						
	$T_{\min}^{P}$	${℘}_{TP}$	${℘}_{T}$	$\sigma_{P\min}/\times 10^{5}$	${℘}_{KP}$	${℘}_{K}$	*S_struc_*
**A**	379.3879	0.9791	0.9055	5.6152	0.9358	0.3866	0.6321
**B**	359.3879	0.9734	0.8577	5.6152	0.9362	0.3866	0.6012
**C**	377.3452	0.9856	0.9066	5.6155	0.9359	0.3886	0.6294
**CDLR**	412.65	0.94	0.821	5.6053	0.936	0.378	0.6193

**Table 4:** Motion errors on the circle with different elastic elements in the BM cables.

Test	The Motion Errors on the Small Circle with the Radius *R*1						The Motion Errors on the Large Circle with the Radius *R*2					
	*X*-Axis		*Y*-Axis		*Z*-Axis		*X*-Axis		*Y*-Axis		*Z*-Axis	
	Mean	Max	Mean	Max	Mean	Max	Mean	Max	Mean	Max	Mean	Max
**A**	1.733	6.55	0.33	5.70	0.123	1.044	2.03	11.025	1.178	11.72	1.38	2.41
**B**	2.088	17.37	8.62	7.52	0.29	1.72	2.35	22.48	9.13	34.02	1.97	3.59
**C**	1.083	11.02	3.52	6.27	0.175	1.33	1.52	15.65	4.17	25.9	1.46	2.59

## References

[1] Bacci M.L. (2017). A Concise History of World Population.

[2] Kanasi E., Ayilavarapu S., Jones J. (2016). The aging population: Demographics and the biology of aging. Periodontology 2000.

[3] Wang Y.L., Wang K.Y., Zhao W.Y., Wang W.L., Han Z., Zhang Z.X. (2019). Effects of single crouch walking gaits on fatigue damages of lower extremity main muscles. J. Mech. Med. Biol..

[4] De-La-torre R., Oña E.D., Balaguer C., Jardón A. (2020). Robot-aided systems for improving the assessment of upper limb spasticity: A systematic review. Sensors.

[5] Sanjuan J.D., Castillo A.D., Padilla M.A., Quintero M.C., Gutierrez E.E., Sampayo I.P., Hernandez J.R., Rahman M.H. (2020). Cable driven exoskeleton for upper-limb rehabilitation: A design review. Robot. Auton. Syst..

[6] Basteris A., Nijenhuis S.M., Stienen A.H.A. (2014). Training modalities in robot-mediated upper limb rehabilitation in stroke: A framework for classication based on a systematic review. J. NeuroEng. Rehabil..

[7] Chen S.H., Lien W.M., Wang W.W., Lee G.D., Hsu L.C., Lee K.W., Lin S.Y., Lin C.H., Fu L.C., Lai J.S. (2016). Assistive control system for upper limb rehabilitation robot. IEEE Trans. Neural Syst. Rehabil. Eng..

[8] Ugurlu B., Nishimura M., Hyodo K., Kawanishi M., Narikiyo T. (2015). Proof of concept for robot-aided upper limb rehabilitation using disturbance observers. IEEE Trans. Hum. Mach. Syst..

[9] Jin X., Prado A., Agrawal S.K. (2018). Retraining of Human Gait—Are Lightweight Cable-Driven Leg Exoskeleton Designs Effective?. IEEE Trans. Neural Syst. Rehabil. Eng..

[10] Kino H., Yoshitake T., Wada R., Tahara K., Tsuda K. (2018). 3-DOF planar parallel-wire driven robot with an active balancer and its model-based adaptive control. Adv. Robot..

[11] Wang Y.L., Wang K.Y., Wang W.L., Yin P.C., Han Z. (2019). Appraise and analysis of dynamical stability of cable-driven lower limb rehabilitation training robot. J. Mech. Sci. Tech..

[12] Qian S., Zi B., Shang W.-W., Xu Q.S. (2018). A review on cable-driven parallel robots. Chin. J. Mech. Eng..

[13] Zou Y.P., Wang N., Wang X.Q., Ma H.Z., Liu K. (2019). Design and Experimental Research of Movable Cable-Driven Lower Limb Rehabilitation Robot. IEEE Access.

[14] Zou Y.P., Liu K., Wang N., Li J.Q., Geng X.H., Chang K.T. Design and Optimization of Movable Cable-Driven Lower-Limb Rehabilitation Robot. Proceedings of the 3rd International Conference on Advanced Robotics and Mechatronics (ICARM).

[15] Wang Y.L., Wang K.Y., Zhang Z., Han Z., Wang W.L. (2020). Analysis of dynamical stability of rigid-flexible hybrid-driven lower limb rehabilitation robot. J. Mech. Sci. Tech..

[16] Wang K.-Y., Yin P.-C., Yang H.-P., Tang X.-Q. (2018). The man-machine motion planning of rigid-flexible hybrid lower limb rehabilitation robot. Adv. Mech. Eng..

[17] Barbosa A.M., Carvalho J.C.M., Goncalves R.S. (2018). Cable-driven lower limb rehabilitation robot. J. Braz. Soc. Mech. Sci. Eng..

[18] Zhao T., Zi B., Qian S., Zhao J.H. (2020). Algebraic Method-Based Point-to-Point Trajectory Planning of an Under-Constrained Cable-Suspended Parallel Robot with Variable Angle and Height Cable Mast. Chin. J. Mech. Eng..

[19] Scalera L., Gallina P., Seriani S., Gasparetto A. (2018). Cable-Based Robotic Crane (CBRC): Design and Implementation of Overhead Traveling Cranes Based on Variable Radius Drums. IEEE Trans. Robot..

[20] Seriani S., Gallina P. (2016). Variable radius drum mechanisms. J. Mech. Robot..

[21] Tang X. (2014). An overview of the development for cable-driven parallel manipulator. Adv. Mech. Eng..

[22] Hong H.J., Ali J., Ren L. (2018). A review on topological architecture and design methods of cable-driven mechanism. Adv. Mech. Eng..

[23] Iandolo R., Marini F., Semprini M., Laffranchi M., Mugnosso M., Cherif A., De Michieli L., Chiappalone M., Zenzeri J. (2019). Perspectives and challenges in robotic neurorehabilitation. Appl. Sci..

[24] Wang Y.L., Wang K.Y., Zhang Z.X., Han Z., Zhang Z.X. (2020). Appraisement and Analysis of Dynamical Stability of Under-Constrained Cable-Driven Lower-Limb Rehabilitation Training Robot. Robotica.

[25] Wang Y.L., Wang K.Y., Zhang Z.X. (2020). Design, comprehensive evaluation, and experimental study of a cable-driven parallel robot for lower limb rehabilitation. J. Braz. Soc. Mech. Sci. Eng..

[26] Qian S., Bao K.L., Zi B., Zhu W.D. (2020). Dynamic Trajectory Planning for a Three Degrees-of-Freedom Cable-Driven Parallel Robot Using Quintic B-Splines. J. Mech. Des..

[27] Wei H.L., Qui Y.Y., Sheng Y. (2019). On the cable pseudo-drag problem of cable-driven parallel camera robots at high speeds. Robotica.

[28] Wei H.L., Qui Y.Y., Sheng Y. (2016). Motion stable control for cable-driven parallel camera robots with high speeds. J. Xidian Univ..

[29] Boschetti G., Carbone G., Passarini C. (2019). Cable failure operation strategy for a rehabilitation cable-driven robot. Robotics.

[30] Zi B., Yin G.C., Li Y., Zhang D. Kinematic Performance Analysis of a Hybrid-Driven Waist Rehabilitation Robot. Proceedings of the 2nd International Conference on Mechatronics and Robotics Engineering (ICMRE).

[31] Ghobj S., Akl A., El-Farr A., Ayyash M., Abu-Khalaf J. Mechanical Design for a Cable Driven Upper Limb Exoskeleton Prototype Actuated by Pneumatic Rubber Muscles. Proceedings of the International Conference on Research and Education in Mechatronics (REM).

[32] Zi B., Yin G., Zhang D. (2016). Design and Optimization of a Hybrid-Driven Waist Rehabilitation Robot. Sensors.

[33] Wang Y.L., Wang K.Y., Zhang Z.X., Mo Z.J. (2020). Control strategy and experimental research of a cable-driven lower limb rehabilitation robot. Proc. Inst. Mech. Eng. Part C J. Eng. Mech. Eng. Sci..

[34] Chen Q., Zi B., Sun Z., Li Y., Xu Q.S. (2019). Design and Development of a New Cable-Driven Parallel Robot for Waist Rehabilitation. IEEE-ASME Trans. Mechatron..

[35] Plooij M., Keller U., Sterke B., Komi S., Vallery H., Von Zitzewitz J. (2018). Design of RYSEN: An Intrinsically Safe and Low-Power Three-Dimensional Overground Body Weight Support. IEEE Robot. Autom. Lett..

[36] Liu X., Qiu Y.Y., Sheng Y. (2011). Analysis on the static stiffness of wire-driven parallel manipulators. J. Mech. Eng..

